# The term CAKUT has outlived its usefulness: the case for the defense

**DOI:** 10.1007/s00467-022-05678-z

**Published:** 2022-07-22

**Authors:** Nine V. A. M. Knoers

**Affiliations:** Department of Genetics, University Medical Center Groningen, Groningen, The Netherlands

**Keywords:** CAKUT, Chronic kidney disease, Shared etiology, Variable expressivity, Reduced penetrance, Genetic counseling

## Abstract

Congenital anomalies of the kidney and urinary tract form a spectrum of congenital structural disorders that are generally known under the term CAKUT. The term CAKUT was introduced 20 years ago and has been used extensively in literature since. Prof. Woolf has made a plea for abandoning this term in his “case for the prosecution.” Here, I advocate for the continued use of CAKUT as an umbrella term for these related congenital kidney and urinary tract abnormalities. I explain why the term CAKUT accurately and usefully defines this group of related structural disorders with prenatal origin and why it makes sense to continue grouping these disorders given accumulating evidence for shared etiology of CAKUT phenotypes and the importance of grouping CAKUT phenotypes in genetic counseling.

## Introduction

The term Congenital Anomalies of the Kidney and Urinary Tract (CAKUT) refers to a wide range of structural abnormalities resulting from a perturbation in the embryonic development of the kidney and urinary tract [1, 2]*,* a complex orchestrated process that involves reciprocal interaction between the ureteric bud and the metanephric mesenchymal tissue [3, 4]. CAKUT constitute ~ 20–30% of all congenital malformations, and their prevalence has been estimated to range between 3 and 6 per 1000 births [5–7]. In ERKReg ( https://www.erknet.org/patients-registry), a web-based registry for all patients with rare kidney diseases of the European Rare Kidney Disease Reference Network “ERKNet” (https://www.erknet.org/), CAKUT are the predominant disease group in children, and an important cause of chronic kidney disease (CKD) in children and adolescents [8]. The 2016 European Society for Paediatric Nephrology (ESPN)/European Renal Association (ERA) registry reported CAKUT in approximately 30% of children with kidney failure [9]. However, CAKUT are also an important disease group in adult CKD patients. In older literature, CAKUT are mentioned as the cause of CKD stage 5 in approximately 5% of adults on kidney replacement therapy, but this may well be an underestimation [10].

The phenotypic spectrum of CAKUT is very broad and includes variable degrees of parenchymal defects of the kidney (such as agenesis, hypoplasia, dysplasia, or multicystic dysplastic kidney), upper urinary tract defects (such as uretero-pelvic junction obstruction, obstructive and/or refluxing megaureter, or vesicoureteral reflux), and lower urinary tract obstruction (such as posterior urethral valves). The severity of CAKUT also varies greatly, from benign conditions such as ectopic kidney to lethal diseases like bilateral kidney agenesis or bilateral multicystic dysplastic kidney [11].

In most cases, CAKUT present as isolated conditions, but there are numerous rare genetic syndromes in which CAKUT are associated with various extrarenal phenotypes. Examples include the renal coloboma syndrome (RCS), an autosomal dominant disorder characterized by both ocular and kidney anomalies (OMIM #120330) [12], and the autosomal dominant branchio-oto-renal (BOR) syndrome characterized by the combination of kidney anomalies, branchial fistula, and hearing impairment (OMIM #113650) [13].

Since its introduction in 1998 by the group of Ishikawa [14], the term CAKUT has been adopted by medical and scientific communities and used extensively in literature. In a paper published earlier in this journal, Professor Adrian Woolf made a plea for abandoning the term [15]. Here, I do the opposite, advocating instead for the continued use of CAKUT as an umbrella term for these related congenital kidney and urinary tract abnormalities. I start explaining why the term CAKUT usefully and accurately defines this group of related structural disorders with prenatal origin. I then explain why it makes sense to continue to group these related disorders, based on two arguments: (1) accumulating evidence for the shared etiology of CAKUT phenotypes and (2) the importance of grouping CAKUT phenotypes in genetic counseling.

## Why CAKUT is a useful and accurate term

The term “Congenital Anomalies of the Kidney and Urinary tract” (acronym CAKUT) best describes which disorders belong to this group and also makes clear which disorders do not, but it is worth breaking down the term to understand why this is the case. To start, the term “congenital” refers to “present at birth” and of prenatal origin. However, congenital does not necessarily mean genetic, as other factors can also be causative. For instance, environmental and/or maternal factors can also lead to congenital defects, as we know from the congenital limb anomalies caused by fetal thalidomide exposure and from maternal diabetes as a risk factor for causing CAKUT. In other words, not all congenital disorders are genetic and not all genetic disorders are congenital. The Mendelian (monogenic) disorder polycystic kidney disease (PKD), for instance, can be a congenital disorder, as it often is in ARPKD, but in most cases is not. The same holds true for other Mendelian kidney disorders, such as nephronophthisis and steroid-resistant nephrotic syndrome. In several reported patients, mutations in some of the involved genes (i.e., *NPHP1*, *NPHP4*, *PKHD1*) manifest as phenocopies of CAKUT, but the related Mendelian disorders (nephronophthisis, ARPKD) do not belong to CAKUT [16, 17].

The term “anomaly” refers to “something that deviates from normal,” and the same holds true for the term “abnormality.” In fact, both can be used interchangeably, although there might be a subtle difference in their interpretation, with abnormality having a more negative implication. Therefore, several years ago, the wording behind CAKUT was changed from “congenital abnormalities of the kidney and urinary tract” to “congenital anomalies of the kidney and urinary tract,” consistent with the wording of other congenital birth defects such as those of the nervous system, limbs, and gastrointestinal system.

Together, the term “congenital anomalies of the kidney and urinary tract” (acronym CAKUT) correctly describes a group of frequently occurring related structural disorders that have a prenatal origin (disturbed nephrogenesis) and are therefore “present at birth” (congenital) and can be discriminated from many Mendelian kidney disorders, which may or may not be congenital.

The term CAKUT has been used for more than 20 years, mainly in pediatric nephrology; CAKUT are of great relevance in daily clinical practice of pediatric nephrologists due to their high prevalence, and because they form the leading cause of CKD in children. Many children with CAKUT now survive to reach adulthood due to substantial advances in early (prenatal) detection, surgical treatment, and improvements in neonatal and pediatric intensive care and general (conservative) management of pediatric CKD both before and after transplantation. The growing number of children with CAKUT who now transition into adult nephrology care explains why nephrologists treating adults are also becoming increasingly aware of CAKUT among their CKD patients. Moreover, with the more frequent use of genetic testing in the diagnosis of patients with adult-onset CKD, it has become clear that CAKUT are also common among these patients [18, 19].

In addition to growing awareness of CAKUT among medical specialists, many organizational initiatives have been undertaken in previous years to encourage and support expert diagnostics and care, disease registration, patient education, and advocacy and research. All these initiatives use CAKUT as an umbrella term to express that the congenital anomalies within CAKUT are seen as a group of related disorders that deserve a holistic approach. Examples of these projects include CAKUT expertise centers, CAKUT disease registries (e.g., ERKreg), CAKUT working groups (e.g., in ESPN (http://www.espn-online.org/cakut-uti-bladder-dysfunction/)), and CAKUT diagnostic groups in patient advocacy organizations (e.g., Kidney patients foundation Netherlands).

## Accumulating evidence for the shared etiology of CAKUT phenotypes

While the etiological landscape of CAKUT is very complex and only partly elucidated, a broadly accepted etiological hypothesis is that genes essential for kidney development are subject to genetic, environmental, and epigenetic modifications that could disrupt their regulation and result in increased susceptibility to CAKUT [1]. Familial clustering of CAKUT suggests an important genetic contribution to its etiology [20]. In a minority of CAKUT cases, the influence of a genetic defect is indeed the determining causal factor in the disorder. These “monogenic” forms of CAKUT are mostly explained by pathogenic single gene variants (10–15% of cases). The genes most frequently involved in monogenic CAKUT are *PAX2*, *HNF1B*, and *EYA1* [21], with mutations in other genes each accounting for only a small percentage of cases [22]. In recent years, pathogenic variants in more than 50 genes have been shown to cause isolated and syndromic CAKUT, following an autosomal dominant or, less frequently, a recessive or X-linked model of inheritance [22, 23]. This broad genetic heterogeneity is the main reason why large next-generation sequencing–based multi-gene panels that include all known CAKUT genes are now used for genetic diagnostic testing in patients.

Pathogenic copy number variants (CNVs) are a second important cause of monogenic CAKUT. CNVs are structural variations in the genome of an individual in the form of gains (duplications) or losses (deletions) of DNA fragments. These CNVs range in size from 1 kb to several megabases (Mb). CNVs, which occur widely in our genomes, are an important source of both normal and pathogenic genetic variation and have been shown to be involved in a wide variety of human diseases, including developmental disorders like CAKUT [24]. Examples of CNVs relevant for CAKUT are the 22q11.2 microdeletion and the 17q12 microdeletion [25, 26]. In a recent study, known CAKUT-causing CNVs were identified in 4.1% of a series of 2824 CAKUT cases, and novel CNVs were associated with an additional 2% of cases [21].

**Fig. 1:**
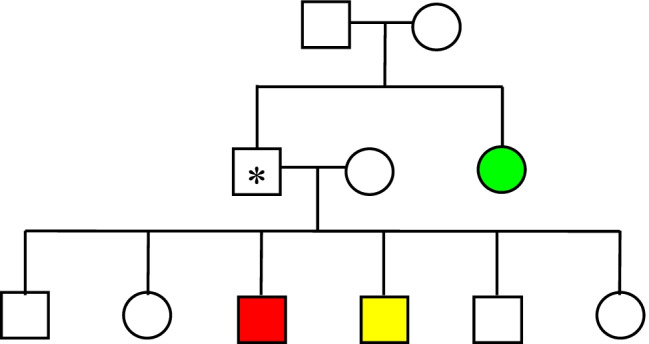
Schematic example of intrafamilial variability in CAKUT, showing both variable phenotypes (different colors) and reduced penetrance (indicated by *)

Monogenic CAKUT are characterized by significant phenotypic variability, including both intra-individual and inter-individual variability. Intra-individual variability can be seen in the co-occurrence of different anomalies within the same individual. For instance, meta-analyses have shown that ~ 1 in 3 cases with unilateral kidney agenesis or multicystic dysplastic kidneys has vesicoureteral reflux (VUR) or ureteropelvic junction obstruction on the contralateral side [27, 28]. In addition, discordant phenotypes have been reported in monozygotic twins, who have exactly the same genotype [29, 30]. Inter-individual variability is obvious from the observation that identical pathogenic variation can result in different CAKUT sub-phenotypes and in variable severity, with or without extrarenal features, even within the same family, demonstrating the complex genotype–phenotype correlation in CAKUT. In families with monogenic CAKUT, both variable phenotypes and reduced penetrance have been described [31, 32] (Fig. 1). Although the exact determinants of penetrance and expressivity in monogenic disorders are not yet known, a growing body of research supports an important contribution to monogenic risk via additional common small-effect genetic variants, the so-called polygenic background. The impact of polygenic background on penetrance and expressivity has been shown, for instance, in familial hypercholesterolemia, hereditary breast and ovarian cancer, hereditary colon cancer, and monogenic metabolic conditions [33, 34]. Whether this also holds true for CAKUT remains to be elucidated. In addition, environmental and epigenetic factors may directly affect the CAKUT phenotype [1]. Whatever the cause of the variable expressivity, the observation of different CAKUT phenotypes in families argues for approaching the congenital anomalies of the kidney and urinary tract as a group of strongly related disorders that can occur together in the same family and are primarily caused by the same defect, i.e., a rare pathogenic gene variant. This shared etiological basis forms a strong argument to maintain these phenotypes within one collective group, best described under the term CAKUT.

For the majority of cases, the etiology of CAKUT is still unknown. In these cases, there is no evidence of a rare large-effect pathogenic gene variant as the primary determinant of the disorder, and the phenotype is most likely caused by the concerted action of a few (oligogenic) or many (polygenic) small-effect genetic variants in combination with environmental and/or epigenetic influences [1, 35]. To identify these common variants, large-scale investigations such as genome-wide associations studies (GWASs) in large cohorts of cases and population-matched controls are needed. To date, GWASs for CAKUT phenotypes have been few, and most were underpowered to identify genetic risk loci with genome-wide significance [36, 37]. Recently, however, in a GWAS meta-analysis of VUR in seven genetically matched case–control GWAS cohorts of European ancestry, Verbitsky et al. identified three significant genetic risk loci with notably large effects on the phenotype. Interestingly, these risk loci encompass canonical developmental genes such as *WDPCP* and *WNT5A* that are expressed in the developing urogenital tract [38]. To understand their real impact, these promising findings need to be replicated in independent cohorts. In the coming years, the Dutch ArtDECO consortium aims to perform a meta-GWAS in a combined cohort of > 10,000 genotyped individuals with CAKUT, including all CAKUT sub-phenotypes. The results of such a meta-analysis could also answer the question of whether the sub-phenotypes in complex CAKUT also share primary causative factors.

Although the knowledge about environmental factors that increase the risk of CAKUT remains limited, several studies have found associations between the occurrence of CAKUT and maternal factors, such as diet, substance use or medications, or maternal conditions like overweight and diabetes [2, 39]*.* Although there is still much conflicting data about the role of these maternal factors, maternal diabetes and maternal overweight have been consistently identified as environmental hazards in the etiology of CAKUT. Maternal diabetes, in particular, has been linked to a wide range of CAKUT phenotypes, again pointing to a common etiological basis for these congenital anomalies.

Studies on the influence of epigenetics in the etiology of CAKUT are in an early phase. Epigenetic modifications are molecular “switches” capable of turning genes on and off or of fine-tuning gene expression, which can result in a change in phenotype without a change in genotype. The main mechanisms involved are DNA methylation, histone modifications, chromatin remodeling, and non-coding RNA-based gene regulation. In recent years, evidence has been found, mainly in animal studies, that epigenetic factors play an important role in kidney development and are therefore likely to be involved in the etiology of CAKUT [40]. For instance, tissue-specific loss of the methyltransferase *Dnmt1* (responsible for methylation of newly synthesized DNA) during kidney development in mice was associated with a marked decrease in global methylation levels and expression of early embryonic genes and non-renal lineage genes, and this resulted in severe hypodysplastic kidneys with undifferentiated nephrons [41]. These findings show the importance of *Dnmt1* for kidney development. Conditional inactivation of a miRNA-activating enzyme, Dicer1, in the developing mouse kidney results in severe kidney hypoplasia, indicating that there is also an essential role for non-coding RNA-based gene regulation in mammalian kidney development [42]. In humans, however, mutations in *DICER1* are not associated with CAKUT but rather with a pleiotropic tumor predisposition syndrome that can include cystic nephroma, a rare benign kidney tumor [43].

Many more studies are needed to understand the complex interactions between (small-effect) common genetic variants and environmental and epigenetic causes to unravel the etiology of CAKUT in the majority of cases and learn whether, and if so how, specific interactions are associated with specific sub-phenotypes. For now, based on the observations that rare large-effect pathogenic variants and environmental factors are both associated with a wide range of differing phenotypes and that expressivity and penetrance in monogenic CAKUT can vary even among family members carrying the same pathogenic variant, we have sufficient arguments to conclude that the different CAKUT sub-phenotypes share primary causative factors and that we should consider these anomalies to be strongly related. This shared etiology argues strongly for maintaining the different sub-phenotypes within one collective grouping under the umbrella term CAKUT.

## The importance of grouping CAKUT phenotypes in genetic counseling

The genetic heterogeneity, reduced penetrance, and phenotypic variability of CAKUT make genetic counseling exceptionally difficult and challenging. Parents of a child with a CAKUT sub-phenotype and a pathogenic variant in a known CAKUT gene are interested not only in recurrence risk, but also—and more importantly—in the expected phenotype of the potentially affected next child. However, the known intrafamilial variability in monogenic CAKUT hampers predictions of the phenotype of a potentially affected next child, making it crucial that we help parents understand that the phenotype of their affected child is part of a spectrum of congenital malformations, together called CAKUT, and that there is a chance that the next child may have a different, potentially more severe, phenotype than the proband.

The intrafamilial variability is also relevant in light of prenatal diagnostics. Although it is technically possible to perform prenatal genetic testing early in pregnancy (11 weeks) in a family with a known monogenic cause of CAKUT, the variable expressivity poses significant difficulties for clinicians looking to provide prognostic information on mutation-positive fetuses that have yet to demonstrate clinical signs of the condition. For the prospective parents, the extremely broad prognosis makes it difficult to make decisions regarding continuation or termination of a genetically affected pregnancy. Although the kidneys can be visualized by ultrasonography at 11–12 weeks of pregnancy, not all phenotypes within the CAKUT spectrum can be detected at this early gestational age. This means that serial ultrasonography is often necessary in at-risk pregnancies (with fetuses carrying the familial pathogenic variant) to determine how the mutation has affected the fetus. In some cases, that question remains unanswered in the prenatal period.

Until we can really understand the underlying factors that determine intrafamilial variability and reduced penetrance, and have tools that can predict exact phenotypes based on this knowledge, it is important to counsel patients and parents on the full spectrum of the congenital anomalies of the kidney and urinary tract, which we can describe under the umbrella term CAKUT. Using the term CAKUT can also help parents and other counselees find useful disease information or find others with similar conditions in patient advocacy organizations.

## Conclusions

In summary, I have presented arguments for why the term CAKUT should continue to be used as an umbrella term. I discussed why the description “congenital anomalies of the kidney and urinary tract” (acronym CAKUT) usefully and accurately describes which disorders fall in that group. I then laid out two arguments in favor of maintaining a classification that groups these related structural anomalies. Firstly, there is evidence from studies of monogenic CAKUT and those investigating the environmental factors involved in CAKUT that the same causative factor is responsible for different sub-phenotypes of CAKUT. This shared etiology forms a strong argument to consider these sub-phenotypes as a spectrum of strongly related congenital malformations belonging to one collective group that we can best describe using the term CAKUT. Secondly, grouping these phenotypes remains important for genetic counseling, where patients and parents need to be informed about the full spectrum of CAKUT abnormalities as there is a fair chance that a subsequent child will have a different phenotype than the proband. While CAKUT, as Prof. Woolf states [15], may not be a useful term or concept for practitioners whose work requires specificity, such as urologists, it remains a useful and necessary term for practitioners such as clinical geneticists whose work requires explaining a condition with a spectrum of possible phenotypes and for the patients they are treating. Furthermore, given growing evidence for shared etiology in CAKUT conditions, it may be a term that remains needed in the near future.
